# Supplementary material for: Seroreversion to Chlamydia trachomatis Pgp3 antigen among children in a hyperendemic region of Amhara, Ethiopia
Source: medRxiv. 2023 Dec 21:2023.02.04.23285360. Originally published 2023 Feb 7. Preprint. [Version 2] doi: 10.1101/2023.02.04.23285360 (PMC9934712; doi:10.1101/2023.02.04.23285360)
Supplement: Supplement 1 [file NIHPP2023.02.04.23285360v2-supplement-1.pdf]

medRxiv preprint doi: https://doi.org/10.1101/2023.02.04.23285360; this version posted December 21, 2023. The copyright holder for this preprint (which was not certified by peer review) is the author/funder, who has granted medRxiv a license to display the preprint in perpetuity. This article is a US Government work. It is not subject to copyright under 17 USC 105 and is also made available for use under a CC0 license.

SUPPLEMENTAL MATERIAL

Supplementary Table 1. Summary of child characteristics in the longitudinal cohort at enrollment, number of measurements and annual periods contributed to the analysis by follow-up status. Children who did not contribute to the analysis (Excluded) did not have two adjacent serology measurements in annual surveys. Children who had complete follow-up were measured four times and contributed to three annual risk periods.

	Excluded (N=290)	Partial Follow-up (N=716)	Complete Follow-up (N=505)	Overall (N=1,511)
**Age, years**				
Mean (SD)	2.81 (2.19)	2.88 (1.41)	3.00 (1.32)	2.91 (1.56)
**Pgp3 IgG (MFI-bg)**				
Negative	196 (67.6%)	494 (69.0%)	349 (69.1%)	1,039 (68.8%)
Positive	94 (32.4%)	222 (31.0%)	156 (30.9%)	472 (31.2%)
**Trachomatis Follicular (TF)**				
Negative	91 (31.4%)	279 (39.0%)	179 (35.4%)	549 (36.3%)
Positive	109 (37.6%)	360 (50.3%)	306 (60.6%)	775 (51.3%)
Missing	90 (31.0%)	77 (10.8%)	20 (4.0%)	187 (12.4%)
***C. trachomatis* PCR**				
Negative	182 (62.8%)	568 (79.3%)	442 (87.5%)	1,192 (78.9%)
Positive	27 (9.3%)	107 (14.9%)	60 (11.9%)	194 (12.8%)
Missing	81 (27.9%)	41 (5.7%)	3 (0.6%)	125 (8.3%)
**First survey measured**				
0 (baseline)	176 (60.7%)	397 (55.4%)	505 (100%)	1078 (71.3%)
12 m	51 (17.6%)	282 (39.4%)	0 (0%)	333 (22.0%)
24 m	39 (13.4%)	37 (5.2%)	0 (0%)	76 (5.0%)
36 m	24 (8.3%)	0 (0%)	0 (0%)	24 (1.6%)
**Measurements (num.)**				
1	173 (59.7%)	0 (0%)	0 (0%)	173 (11.4%)
2	117 (40.3%)	248 (34.6%)	0 (0%)	365 (24.2%)
3	0 (0%)	468 (65.4%)	0 (0%)	468 (31.0%)
4	0 (0%)	0 (0%)	505 (100%)	505 (33.4%)
**Annual risk periods contributed**				
0	290 (100%)	0 (0%)	0 (0%)	290 (19.2%)
1	0 (0%)	519 (72.5%)	0 (0%)	519 (34.3%)
2	0 (0%)	197 (27.5%)	0 (0%)	197 (13.0%)
3	0 (0%)	0 (0%)	505 (100%)	505 (33.4%)

medRxiv preprint doi: https://doi.org/10.1101/2023.02.04.23285360; this version posted December 21, 2023. The copyright holder for this preprint (which was not certified by peer review) is the author/funder, who has granted medRxiv a license to display the preprint in perpetuity. This article is a US Government work. It is not subject to copyright under 17 USC 105 and is also made available for use under a CC0 license.

Supplementary Table 2. Sample size, age distribution, and seroprevalence in the longitudinal cohort, by month of the study.

Study month	Total number of children with serology samples	Median age in months (IQR)	Seroprevalence (%)
0	1,078	36 (24-48)	31.4
12	1,155	48 (35-60)	37.6
24	1,015	60 (47-72)	42.2
36	1,079	72 (60-96)	51.3

medRxiv preprint doi: https://doi.org/10.1101/2023.02.04.23285360; this version posted December 21, 2023. The copyright holder for this preprint (which was not certified by peer review) is the author/funder, who has granted medRxiv a license to display the preprint in perpetuity. This article is a US Government work. It is not subject to copyright under 17 USC 105 and is also made available for use under a CC0 license.

Supplementary Table 3. Seroconversion rate (SCR) and seroreversion rate (SRR) estimates overall and by subgroups. Children who were Pgp3 negative (–) at the beginning of a one-year period were at risk for seroconversion, and those who were Pgp3 positive (+) were at risk for seroreversion.

	Seroconversion Rate*				Seroreversion Rate*			
	N child Pgp3-	Person-years at risk	Incident conversion	SCR	N child Pgp3+	Person-years at risk	Incident conversion	SRR
**Overall**	1542	1432.5	219	15.3	886	875.0	22	2.5
**Study Period**								
0–12m	566	522.5	87	16.7	256	251.5	9	3.6
12–24m	536	501.5	69	13.8	308	303.0	10	3.3
24–36m	440	408.5	63	15.4	322	320.5	3	0.9
**Child age**								
0 – 5 y	1310	1214.0	192	15.8	698	688.5	19	2.8
6 – 9 y	231	217.5	27	12.4	187	185.5	3	1.6
**IgG level†**								
[0.0, 3.0]	1536	1427.0	218	15.3	0	0.0	0	--
(3.0, 3.5]	6	5.5	1	18.2	47	40.0	14	35.0
(3.5, 4.0]	0	0.0	0	--	92	89.5	5	5.6
(4.0, 5.0]	0	0.0	0	--	747	745.5	3	0.4

* SCR: Seroconversion rate per 100 person-years, SRR: Seroreversion rate per 100 person-years
† Pgp3 IgG level at the start of the one-year period, in $\log_{10}$ units of Median Florescence Intensity minus background (MFI-bg) measured on the Luminex platform.
